# The effects of antenatal education and telephone counseling on childbirth fear of nulliparous women and their attitudes toward childbirth: a randomized controlled trial

**DOI:** 10.1590/1806-9282.20241147

**Published:** 2025-03-17

**Authors:** Burcin Bektas Pardes, Gulten Guvenc

**Affiliations:** 1 University of Health Sciences Turkey, Gulhane Faculty of Nursing – Ankara, Turkey.

**Keywords:** Nursing, Antenatal education, Counseling, Attitude, Nulliparity, Childbirth, Fear

## Abstract

**OBJECTIVE:**

The aim of this study was to determine the effects of antenatal education and telephone counseling given to nulliparous women regarding their childbirth fear and attitudes toward childbirth.

**METHODS:**

The study design is a prospective, randomized controlled trial. The intervention group received antenatal education and telephone counseling between the 28th and 34th gestational weeks. Data were collected using the Personal Information Questionnaire, Wijma Delivery Expectancy/Experience Questionnaire (WDEQ) Version A, WDEQ Version B, and Childbirth Attitudes Questionnaire.

**RESULTS:**

In terms of sociodemographic and obstetric characteristics, both groups were similar to each other. The mean WDEQ-A and Childbirth Attitudes Questionnaire scores of the intervention and control groups after receiving antenatal education and telephone counseling were 23.78±17.68 and 42.90±24.87 and 21.70±7.29 and 31.71±11.11, respectively. The childbirth fear and childbirth attitude of the intervention group that received antenatal education and counseling were statistically lower than the control group (p=0.001). The mean WDEQ-B scores were 22.82±24.09 and 97.18±32.57, respectively, in the intervention and control groups during the postpartum period. The postpartum childbirth fear of the intervention group was found to be extremely lower than the control group (p=0.001).

**CONCLUSION:**

Imparting antenatal education and counseling on childbirth fear is an effective method to reduce childbirth fear and the negative attitudes toward childbirth in nulliparous women.

**Registration number of the clinical trial:**

The study was registered at the ClinicalTrials.gov Protocol Registration and Results System (protocol ID number: NCT04173351).

## INTRODUCTION

Pregnancy is an important period in a woman's life, during which they experience biological, physiological, emotional, and social changes to adapt to maternity. Pregnancy may be a source of happiness, satisfaction, and self-fulfillment but may also lead to anxiety and concerns about childbirth^1,2^. The worldwide prevalence of severe childbirth fear in pregnant women is 14%^3^. The prevalence of childbirth fear among Turkish pregnant women was approximately 21%^4^.

Childbirth fear is more prevalent among nulliparous women compared to multiparous women. Knowledge deficit, low self-rated health status, and low self-efficacy levels among nulliparous women are the major reasons for childbirth fear^5^. Childbirth fear and prenatal distress are moderately and positively correlated with each other and for this reason, it is important to support positive childbirth experiences^6^. Antenatal education and counseling focus on the psychological and social aspects of pregnancy and childbirth to reduce pregnant women's childbirth fear, help them develop strategies to cope with their fears and anxieties, and improve their childbirth experience^7^. Pregnant women who receive antenatal education within the context of certain principles consider childbirth a normal and natural event. An increase in the knowledge levels of pregnant women during antenatal and postpartum periods makes a positive contribution to the postpartum health of mothers and infants^8^. Antenatal educational programs are often provided in the obstetric outpatient clinics of hospitals in Turkey. The programs usually consist of two to four sessions. The educational programs focus on physiological and psychological changes during pregnancy; stages of labor; methods of coping with labor pain; problems that may occur during pregnancy, birth, and postpartum period; warning signs during pregnancy and postpartum period; contraception methods; and newborn care. These programs are usually conducted by nurses and midwives. In these educational sessions, PowerPoint presentations, verbal lectures, videos, and demonstrations using infant care materials are used^9^. Antenatal education during the last trimester may decrease childbirth fear^10^. Furthermore, it has been found that pregnant women who received antenatal education had increased tolerance for the uncertainty related to the birthing process, positive birth experience, better self-confidence for a future birth, lower cesarean birth rate and use of epidural anesthesia, and fewer symptoms of postpartum depression^11^. In general, in order to decrease pregnant women's childbirth fear, they should be included in the educational programs^12^.

The fear of childbirth is an important health problem for pregnant women^6^. Since evidence on reduced fear of childbirth due to antenatal education in Turkey is limited, the authors decided to conduct this study. This study aims to determine the effects of antenatal education and telephone counseling given to nulliparous women in the last trimester to reduce their childbirth fear and negative attitudes toward childbirth.

## METHODS

### Research design

**Figure 1:**
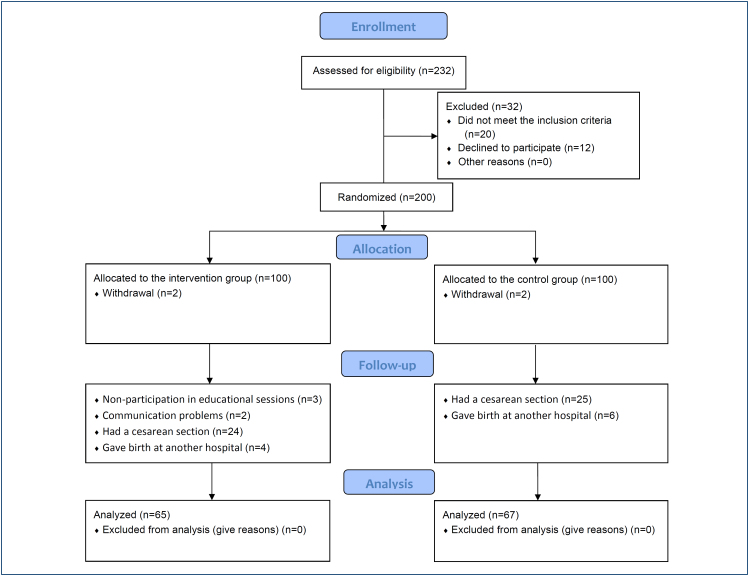
CONSORT flow diagram of the study.

This study was conducted as a prospective, single-center, randomized controlled, two-armed trial. The study was carried out according to CONSORT guidelines (Figure 1), and a clinical trial registration code was obtained (NCT04173351).

### Participants

The population of the study comprised 132 nulliparous women who presented to a training and research hospital in Turkey between February 2016 and January 2017. The sample size was calculated by using the G*Power program. To attain a 99% confidence interval (type I error=0.01 and type II error=0.01) and a power of 0.99, a total of 48 participants were required in each group^13^. The principal investigator conducted the study, provided education and counseling services, and implemented data collection instruments. Voluntary participants who met the inclusion criteria were informed about the aim and scope of the study. After obtaining their consent, eligible pregnant women were allocated in a ratio of 1:1 to the intervention or control group by using a randomization list. The block randomization list for two groups was obtained by using a web-based randomization system.

The inclusion criteria were literate, nulliparous women with a single fetus between the 28th and 34th gestational weeks, those without a high-risk pregnancy or infertility treatment, those who had a vaginal delivery after the pre-test, and those who agreed to participate in the study.

The exclusion criteria were women with a cesarean section and women who were monitored or gave birth at a hospital other than where the present study was conducted.

### Data collection forms

Personal Information Questionnaire (PIQ): The PIQ was developed by researchers based on the literature to obtain data on the sociodemographic and obstetric characteristics of the participants^13^. The questionnaire includes 20 questions and is composed of three parts: sociodemographic characteristics, obstetric history, and a history of antenatal education.

Wijma Delivery Expectancy/Experience Questionnaire (Version A) (WDEQ-A): The WDEQ-A was developed by Klaas and Barbro Wijma in Sweden in 1998. The Cronbach's alpha value of the scale is 0.88 for primiparous and 0.90 for multiparous women. The Turkish validity and reliability of the scale were evaluated by Kızılırmak and Baser. The scale consists of 33 items, which are rated on a 6-point Likert-type scale (0=do not agree; 5=totally agree). The cutoff score of the questionnaire is 85^14^. The Cronbach's alpha value of the scale in this study was 0.94.

Wijma Delivery Expectancy/Experience Questionnaire (Version B) (WDEQ-B): The WDEQ-B was developed by Klaas and Barbro Wijma in 1998 to determine the postpartum pain, feelings, and thoughts of women after childbirth. The Turkish validity and reliability of the scale were determined by Korukcu et al. The questionnaire includes 32 items rated on a 6-point Likert-type scale (0=not at all; 5=extremely). The questionnaire has six subscales: concerns about childbirth fear, loneliness, lack of positive feelings, concerns about childbirth, and concerns about the baby^13^. The Cronbach's alpha value of the scale in this study was 0.98.

Childbirth Attitudes Questionnaire (CAQ): The CAQ was developed by Lowe in 2000 to measure childbirth fear. The Cronbach's alpha value of the scale is 0.83. The Turkish validity and reliability of the scale were determined by Donmez et al. The questionnaire includes 16 items rated on a 4-point Likert-type scale. Higher scores indicate greater fear^15^. The Cronbach's alpha value of the scale in this study was 0.89.

### Content of education

Childbirth Preparation Educational Program: Nulliparous women received a group-based slide presentation on childbirth preparation from the principal researcher at an obstetrics clinic between the 28th and 34th gestational weeks. The educational program covered pregnancy processes, childbirth signs, pre-childbirth preparations, childbirth fear, breathing techniques, positive childbirth experiences, and impacts of vaginal delivery. The programs were completed in two sessions in a single day. The sessions lasted 45 min each with a 15-min break. The participants were given an educational brochure after the sessions.

Telephone Counseling: One of the researchers telephoned the participants in the intervention group 1 week later. This one-to-one counseling aimed to support the expression of feelings, review expectations and feelings, clarify misunderstandings, answer questions, and provide counseling about the demands and points that the nulliparous women wondered about one to one. The telephone counseling took approximately 10-15 min. It was explained that if the participants had any questions during the prenatal period, they could call the principal investigator between 9 am and 9 pm. Participants who called continued to receive telephone counseling.

### Data collection

Data were collected using face-to-face interviews. The pregnant women were asked to complete the PIQ, WDEQ-A, and CAQ between the 28th and 34th gestational weeks. The pregnant women in the intervention group were given antenatal childbirth education and telephone counseling. Participants in both the control and intervention groups were asked to complete the WDEQ-A and CAQ during the 38th and 40th gestational weeks. The WDEQ-B was administered during the first and the second postpartum days in both groups. No intervention was conducted in the control group. Participants in the intervention group received antenatal education and telephone counseling, whereas those in the control group received routine antenatal follow-up.

### Data analysis

The IBM SPSS (Statistical Package for the Social Sciences) 22.0 software was used for data analysis. As descriptive statistics, number (n) and percentage (%) were used for numerical variables and mean ± standard deviation ( $\bar{X}$ ±SD) for categorical variables. The Kolmogorov-Smirnov test was used to analyze the normality of the distribution of continuous variables. Parametric tests were used for the analysis of data with normal distribution. The independent-sample t-test was used for the inter-group comparison of continuous variables, while the paired sample t-test was used for intra-group analysis. A p-value of 0.05 was set for statistical significance.

### Ethical consideration

For studies involving human subjects, the ethical considerations include the following: All procedures followed were in accordance with the ethical standards of the responsible committee on human experimentation (institutional and national) and with the Helsinki Declaration of 1975, as revised in 2008. The ethical approval for the study was obtained from the Ethical Board of the Gulhane Military Medical Academy (No. 50687469-1491-533-15/1648.4-1984). Prior to joining the study, an informed consent form was filled in and written consent was obtained from all the participants. The participants were informed that they could withdraw from the study at any point during the study.

## RESULTS

The mean age of the participants in the intervention and control groups was 26.81±3.80 and 27.76±3.67 years, respectively. More than half of the women in both groups had a university degree and were not working, and more than 80% had planned a pregnancy (Table 1). The mean pre-test WDEQ-A score was 52.00±17.92 in the control group and 58.31±20.26 in the intervention group. The mean post-test WDEQ-A score was 42.90±24.87 in the control group and 23.78±17.68 in the intervention group. The childbirth fear scores of the intervention group after receiving antenatal education and telephone counseling were statistically significant (t=5.034; p=0.001). Besides, a comparison of the pre- and post-test scores of the control and intervention groups showed a statistically significant difference between the pre- and post-test scores of the intervention group (t=6.715; p=0.001) (Table 2).

**Table 1:** Sociodemographic and obstetric characteristics of nulliparous women.

Characteristics		Intervention group (n=65)	Control group (n=67)	t*	P
		$\bar{X}$ ±SD	$\bar{X}$ ±SD		
Age (years)		26.81±3.80	27.76±3.67	-1.115	0.265
Duration of pregnancy (weeks)		31.68±2.23	32.79±2.43	-3.218	0.051
		**n (%)**	**n (%)**	χ^2^	**P**
Educational status					
	Elementary	8 (12.3)	4 (6.0)	2.938	0.401
	High school	14 (21.5)	13 (19.4)		
	University	39 (60.0)	48 (71.6)		
	Master/doctorate	4 (6.2)	2 (3.0)		
Educational status of husband					
	Elementary	1 (1.5)	1 (1.5)	0.088	0.933
	High school	18 (27.7)	19 (29.2)		
	University	41 (61.2)	41 (61.2)		
	Master/doctorate	5 (7.7)	6 (8.0)		
Employment status					
	Employed	19 (29.2)	26 (38.8)	1.346	0.246
	Nonemployed	46 (70.8)	41 (61.2)		
Employment status of husband					
	Employed	65 (100.0)	63 (94.0)	4.002	0.119
	Nonemployed	0 (0.0)	4 (6.0)		
Planned pregnancy					
	Yes	60 (92.3)	54 (80.6)	3.842	0.074
	No	5 (7.7)	13 (19.4)		

$\bar{X}$ : Mean, SD: standard deviation, χ^2^=Pearson's chi-square test.

* Independent-sample t-test.

**Table 2:** Childbirth fear, childbirth attitude, and postpartum childbirth fear scores of the control and intervention groups prior to and after antenatal education and telephone counseling to the intervention group.

Variables	Categories	Intervention group (n=65)	Control group (n=67)	t*	p
		$\bar{X}$ ±SD	$\bar{X}$ ±SD		
WDEQ-A	Pre-test	58.31±20.26	52.00±17.92	-1.953	0.051
	Post-test	23.78±17.68	42.90±24.87	-5.034	**0.001**
	t**/p	-6.715/**0.001**	-2.881/0.084		
CAQ	Pre-test	39.44±9.30	37.59±8.25	1.208	0.229
	Post-test	21.70±7.29	31.71±11.11	-5.663	**0.001**
	t**/p	-6.769/**0.001**	4.615/0.061		
WDEQ-B	Post-test	22.82±24.09	97.18±32.57	-8.951	**0.001**

* Independent-sample t-test.

** Paired sample t-test

$\bar{X}$ : Mean, SD: Standard deviation; WDEQ-A: Wijma Delivery Expectancy/Experience Questionnaire Version A, WDEQ-B: Wijma Delivery Expectancy/Experience Questionnaire Version B, CAQ: Childbirth Attitudes Questionnaire. Statistically significant values are denoted in bold.

The childbirth attitude post-test scores of the intervention and control groups were 21.70±7.29 and 31.71±11.11, respectively. There was a statistically significant difference between the two groups in terms of CAQ scores after imparting antenatal education and telephone counseling to the intervention group (t=-5.663; p=0.001). The pre-test CAQ score of the intervention group decreased from 39.44±9.30 to 21.70±7.29 after imparting antenatal education and counseling, indicating a statistically significant difference between the pre- and post-test scores of the intervention group (t=-6.769; p=0.001) (Table 2).

The mean postpartum childbirth fear scores of the intervention and control groups during the process of childbirth were 22.82±24.09 and 97.18±32.57, respectively. A statistically significant difference was observed between the two groups in terms of childbirth fear during the process (t=-8.951; p=0.001). Furthermore, a statistically significant difference was found between the two groups in terms of the subscales of concerns about childbirth fear, loneliness, lack of positive feelings, concerns about childbirth, and concerns about the baby (p=0.001) (Table 2).

## DISCUSSION

This study aims to determine the effects of antenatal education and telephone counseling given to nulliparous women in the last trimester in order to reduce their childbirth fear and negative attitudes toward childbirth. Nulliparous women have severe childbirth fear, and this fear becomes more intense during the last trimester^16^. Psychoeducation has been found to be an effective approach to reducing childbirth fear. Women who receive psychoeducation will experience more positive pregnancies and birth experiences^17^. In our study, the childbirth fear of the intervention group that received antenatal education and telephone counseling was statistically lower than the control group. In this sense, this study found that antenatal education and telephone counseling on preparations for childbirth and ways to cope with childbirth pain, which were given during the last trimester, significantly reduced the childbirth fear of nulliparous women. In a recent study conducted in Turkey which analyzed the impact of antenatal education on childbirth fear of primiparous women, the mean pre- and post-education WDEQ-A scores of the intervention group were found to be quite low compared to women who did not receive education^18^. Similarly, a single-blinded, randomized controlled trial reported that the post-education WDEQ-A scores of the intervention group were significantly lower than the pre-education WDEQ-A scores^19^. Compared to these studies, the difference between the pre- and post-education childbirth fear scores of the intervention group in our study was higher. This difference may stem from the fact that, unlike the other studies, this study additionally provided one-to-one telephone counseling on preparations for childbirth, ways to cope with childbirth pain, and points that the nulliparous women wondered about.

Childbirth fear has a negative impact on childbirth attitudes by decreasing the satisfaction toward childbirth^20^. In our study, the childbirth attitudes of the intervention group that received antenatal education and telephone counseling were significantly lower than the control group. Similarly, in a recent study on the severe fear of childbirth among primiparous women in Turkey, it was reported that psychoeducation can reduce the fear of childbirth and increase childbirth attitudes^21^.

Childbirth education leads to better childbirth experience and maternal adjustment and fewer depressive symptoms in primiparous women with severe childbirth fear in the postpartum period^22^. In our study, postpartum childbirth fear of the intervention group was extremely lower than the control group. Studies that analyzed the effects of group psychoeducation on nulliparous women with severe childbirth fear found that the postpartum childbirth fear of the intervention group was statistically significantly low^23^.

### Limitations

This study has some limitations that need to be acknowledged. Some of the participants who met the inclusion criteria were excluded from the study since they had a cesarean section. The study was conducted at a single center. Only nulliparous women were included in the antenatal education sessions, and the partners of the pregnant women were not trained. The data obtained were based on the self-reports of pregnant women who participated in the study.

## CONCLUSION

This study found that childbirth fear levels of the nulliparous women decreased after antenatal education and telephone counseling. Antenatal education and telephone counseling provided to nulliparous women during the last trimester decreased their childbirth fear and contributed to their positive attitudes toward childbirth.
